# A Catalog of Natural Products Occurring in Watermelon—*Citrullus lanatus*

**DOI:** 10.3389/fnut.2021.729822

**Published:** 2021-09-14

**Authors:** Maria Sorokina, Kira S. McCaffrey, Erin E. Deaton, Guoying Ma, José M. Ordovás, Penelope M. Perkins-Veazie, Christoph Steinbeck, Amnon Levi, Laurence D. Parnell

**Affiliations:** ^1^Institute for Inorganic and Analytical Chemistry, Friedrich-Schiller University, Jena, Germany; ^2^Universität Konstanz, Konstanz, Germany; ^3^Department of Horticulture, Plants for Human Health Institute, North Carolina State University, Kannapolis, NC, United States; ^4^Nutrition and Genomics Laboratory, Jean Mayer-United States Department of Agriculture (JM-USDA) Human Nutrition Research Center on Aging at Tufts University, Boston, MA, United States; ^5^United States Department of Agriculture (USDA), Agricultural Research Service, U.S. Vegetable Laboratory, Charleston, SC, United States; ^6^United States Department of Agriculture (USDA), Agricultural Research Service, Nutrition and Genomics Laboratory, Jean Mayer-United States Department of Agriculture (JM-USDA) Human Nutrition Research Center on Aging at Tufts University, Boston, MA, United States

**Keywords:** food chemistry, natural products, natural compounds, watermelon, phytochemicals

## Abstract

Sweet dessert watermelon (*Citrullus lanatus*) is one of the most important vegetable crops consumed throughout the world. The chemical composition of watermelon provides both high nutritional value and various health benefits. The present manuscript introduces a catalog of 1,679 small molecules occurring in the watermelon and their cheminformatics analysis for diverse features. In this catalog, the phytochemicals are associated with the literature describing their presence in the watermelon plant, and when possible, concentration values in various plant parts (flesh, seeds, leaves, roots, rind). Also cataloged are the chemical classes, molecular weight and formula, chemical structure, and certain physical and chemical properties for each phytochemical. In our view, knowing precisely what is in what we eat, as this catalog does for watermelon, supports both the rationale for certain controlled feeding studies in the field of precision nutrition, and plant breeding efforts for the development of new varieties with enhanced concentrations of specific phytochemicals. Additionally, improved and comprehensive collections of natural products accessible to the public will be especially useful to researchers in nutrition, cheminformatics, bioinformatics, and drug development, among other disciplines.

## Graphical Abstract

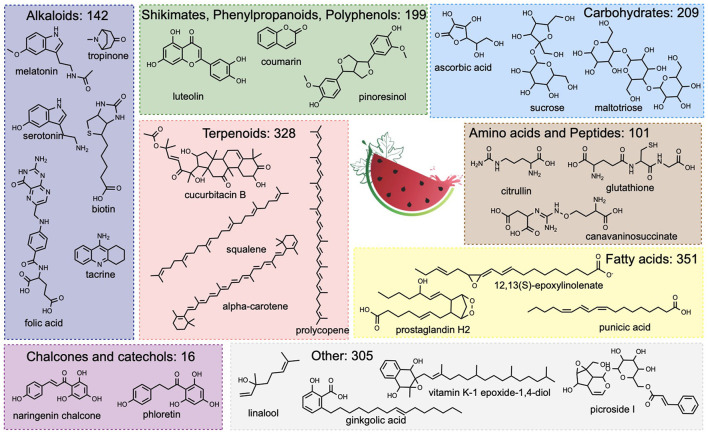

## Introduction

Food is a complex mixture of chemical compounds, often numbering well over a thousand different compounds in any individual food item (1–3). That complexity expands when considering processing (4), the food matrix (5, 6), or byproducts, such as those derived from both human and microbial metabolism (7), as well as taints and off-flavors derived from degradation and packaging (8). Nonetheless, any catalog of the metabolites of food compounds coupled to research on their health effects ought to begin with knowledge of a chemical inventory of what is in the food, and in the forms in which it will be consumed. Here, we focus primarily on watermelon fruit, seed, and rind to provide a comprehensive, publicly accessible list of phytochemicals in watermelon.

Natural product databases generally are at the small end of the size spectrum of chemical databases when compared with the vastly larger PubChem (~110 million compounds) (9) and collections of synthetic compounds numbering in the billions (10). This necessitates a significant and genuine need to build resources for natural products. Existing natural product databases also suffer from missing links between the chemical structures and the organisms that produce them (3). These missing links often result from the standard practice that only newly elucidated structures are reported in scientific journals and then aggregated into public databases (11). Well-known metabolites identified in a newly studied organism or food are not reported. Hence, research programs in nutrition, cheminformatics, and drug development, among other disciplines, will benefit from natural product datasets that are comprehensive in scope and of a design that easily merges with other data.

Regarding human nutrition, knowledge of what is in a food is the basis by which to characterize the health benefits of that food. Those efforts support knowing what to eat to remain healthy (12) and assist in defining the “dark matter” or chemical complexity of nutrition (13, 14). In addition, comprehensive catalogs of the biochemicals present in a crop can stimulate projects in plant breeding and crop improvement, especially when coupled with genome sequencing and other such data streams (15, 16). Thus, to support and then fully implement projects in computational nutrition and cheminformatics research on natural products, and expand capabilities for dietary assessment, we sought to build a comprehensive catalog of compounds naturally occurring in watermelon.

Sweet dessert watermelon (*Citrullus lanatus*) is among the most important vegetable crops grown and consumed throughout the world, with global annual planting of more than 3 million hectares and production of over 100 million tons. China leads the world in watermelon production with an annual output of over 60 million tons. Other top watermelon producing countries are Turkey, India, Brazil, Algeria, Iran, Russia, United States, Egypt, Mexico, Kazakhstan, and Uzbekistan (with an annual production of 3.9, 2.5, 2.3, 2.2, 1.9, 1.8, 1.7, 1.6, 1.3, 1.3, and 1.2 million tons, respectively) (17).

Watermelon belongs to the xerophytic genus *Citrullus*, native to Africa (18). It was domesticated in Africa over 4000 years ago, while sweet dessert watermelons emerged in the Mediterranean region over 2000 years ago (19). It was introduced to India and China by the seventh and tenth centuries, respectively, and to Europe via Moorish Spain in the tenth century. There, watermelon has been cultivated successfully in the warmer Mediterranean regions of the continent. Watermelons were brought to the Americas by European colonists and with the slave trade from Africa during the sixteenth century (19). Today, watermelon is grown in 44 states in the USA, while most production is centered in Texas, Florida, Georgia, and California. Overall, sweet dessert watermelon varieties share a narrow genetic base, indicating a possible origin from a single founder population (20, 21). Those origins, their environments and the growth conditions of current production areas combined with detailed metabolomics will offer insight into origins of favored varieties as well as approaches to use levels of key compounds as quantitative traits for crop improvement.

Watermelon fruits contain a wide range of bioactive compounds, including glycosides, carotenoids, flavonoids, alkaloids, carbohydrates, fatty acids, and essential oils (22). Cucurbitacins, a rather broad family of bitter-tasting compounds in watermelon (23, 24), have drawn interest for their anti-oncogenic pharmacological properties (25). Through many years of evolution, domestication, and selection for desirable qualities, watermelon fruit has undergone significant changes in quality traits, mainly those associated with flesh color and texture, and nutrient and sugar content (26). Developing varieties with desirable fruit characteristics and high nutritional value is a top priority for watermelon breeding programs. Watermelon is a naturally rich source for the non-protein α-amino acid citrulline, which was reported to have antioxidant and vasodilatation activity (27). Citrulline was first isolated from watermelon by the Japanese researchers Yotaro Koga and Ryo Odake in 1914 (28) and further validated in 1930 (29). Lycopene was first reported in watermelon in 1930 (30), and like tomato, watermelon contains high levels of lycopene and other carotenoids with potential benefit for human health (31, 32). The health benefits of some of these compounds are known and continue to be the focus of nutrition research. Yet, interest is growing in documenting the chemical complexity of foods and assessing their impact on human health. The watermelon genome was sequenced, assembled, and annotated in 2019 (21), enabling exploration with bioinformatic tools to elucidate further its nutritive value and identify relevant biochemical pathways to tune the production of compounds of interest.

This manuscript presents a compilation of phytochemicals, linking chemically correct structures to the public resources where they were identified in watermelon and different parts of the plant. The 1,679 natural products that are part of this catalog underwent a curation process, their physicochemical properties were computed with cheminformatics tools, and all data are available at https://watermelon.naturalproducts.net. In this online database, users can freely browse and search for watermelon natural products.

## Materials and Methods

### Data Collection

Scientific articles on watermelon compounds or metabolomics were collected based on queries at PubMed and Agricola, 42 and 22 articles respectively, and supplemented with an additional 15 articles based on careful reading of other articles and 17 via personal communication. We also mined data from watermelon genome (CuCyC, genome v1) (33), and nutrition resources [Food Data Central (34); Phytochemical and Ethnobotanical Database at the USDA (35), PhytoHub (36)], and the LOTUS project (37). The latter aims to catalog documented pairs of natural products and the organisms producing them. Data collection was restricted to *C. lanatus* cultivars, varieties, and grafts. Expert knowledge of the authors directed the cataloging efforts to specific publications. We sought not to incorporate the compounds cataloged at FooDB (https://foodb.ca) and replicate that resource, but do include FooDB identifiers for compounds reported in other sources. Although essential for basic life processes, central metabolites, such as nucleotides, nucleosides, and ubiquitous coenzymes were excluded from the catalog, as these are shared by all living organisms.

### Data Curation

After retrieving the literature, all collected information about the natural products was processed in Java with the Chemistry Development Kit (CDK) (38). For each molecule, the original SMILES were converted to unique and absolute SMILES, implicit hydrogens were tagged accordingly, compound aromaticity was corrected when appropriate, and tautomers and ionization states were standardized. Also, compounds of less than five heavy atoms were discarded. A structure-based compound unification was performed to prevent redundancies within the catalog. This was done using Tanimoto similarity with three different fingerprints, PubChem, Extended, and ECFP fingerprints, and a similarity threshold of 99% between two molecules for three of their fingerprints. Using three different fingerprints is necessary as they do not all perform well on all structure types, in particular for highly redundant monomeric structures like lipids and polysaccharides. The combined fingerprint comparison guarantees that two molecules with a Tanimoto similarity score over 99% with the three approaches are truly identical. The computer code for compound curation, unification, and calculation of features is available on GitHub (https://github.com/mSorok/Watermelon).

### Content of the Catalog

The information on compounds found in watermelon is organized into tables pertinent to two distinct but overlapping disciplines: cheminformatics of natural products and human nutrition. All data are also available on the Watermelon Online website (https://watermelon.naturalproducts.net) with accompanying diverse search functionality. Data presented in these tables include common and alternative names in English, and compound identifiers in major chemical databases: CAS® (Chemical Abstracts Service), KEGG (Kyoto Encyclopedia of Genes and Genomes) (39), HMDB (Human Metabolome Database) (40), PubChem (9), ChEBI (Chemical Entities of Biological Interest) (41), FooDB (https://foodb.ca/), and LipidMaps (https://www.lipidmaps.org) (42). Additionally, provided for each molecule are the molecular formula and weight, together with classic structure representations, such as InChI, original (as from their source), canonical, and absolute SMILES, plus other representations such as Murcko scaffolds (43) (used generally for structure-activity relationship elucidation) and deepSMILES (an adaptation of SMILES for machine-learning purposes) (44). A wide range of molecular descriptors, such as AlogP, topological polar surface, atomic polarizabilities, Zagreb Index, Petitjean number, Kappa shape index, and the Lipinsky rule of five failures, have been computed with the CDK. Chemical pathways, superclasses, and classes were calculated with NPclassifier (https://npclassifier.ucsd.edu/) (45). This dataset is provided in Supplementary Table 1 and is available at https://watermelon.naturalproducts.net.

### AFC Identifiers

Unique identifiers are a convenient means to refer to a compound without ambiguity. However, no single data repository has identifiers for all compounds cataloged here. Thus, we define the “AFC” identifier to represent **A**gricultural Research Service **F**ood **C**ompound and encourage its use in other catalogs. This has been assigned to all entries and serves as a bridge between data resources, the source literature, and across the two tables presented here.

### Nutrition Data

Parallel to cataloging the natural products of watermelon and supporting nutrition research, effort was expended to assemble information, when available, on concentrations of compounds from different parts of the plant. The plant parts for which data are tabulated include (red) flesh, heart tissue, juice, seed, rind, peel, yellow flesh, seedling, leaf, root, other parts of the plant, and detected but plant part not reported. The collected data included the low value in the range, the high value in the range, deviation from those values, and units (assumed to be fresh or wet weight unless noted). This table (Supplementary Table 2) also provides for all compounds the citations to the literature and database sources. This information is archived at the USDA's Ag Data Commons (https://doi.org/10.15482/USDA.ADC/1522862), where updates will be provided.

### Data Analysis

Simple statistical analyses and plots were made with ggplot in R, or Python 3 and the RDkit cheminformatic library for Python (46). The glycosylation analysis was performed with the Sugar Removal Utility (47) and RDkit. The graphical representation of the chemical space covered by the known watermelon natural products was performed with the t-distributed stochastic neighbor embedding (t-SNE), a dimensionality reduction method that captures a large fraction of the overall structural variance across the molecular set. t-SNE was performed with the *scikit-learn* Python 3 library and MACCS fingerprints.

### Genome Mining

The *C. lanatus* genome (accession number GCA_000238415.2) was downloaded from the NCBI Genome on 1 Dec 2020. Online versions of plantiSMASH v.1 (48) and PRISM 3 (49) were used under default parameters to mine this genome for known biosynthetic gene clusters (BGCs) whose products synthesize small molecules such as non-ribosomal peptides (NRP) and polyketides.

### Data Dictionary

The different terms and abbreviations are defined in Supplementary Table 3, and archived at https://doi.org/10.15482/USDA.ADC/1522862.

## Results/Database Description

When writing this manuscript, the cheminformatics catalog of naturally occurring compounds in watermelon contains 1,679 curated molecules (Supplementary Table 1). This set does not include water, dissolved gases, minerals, salts, and common, central metabolism compounds, such as ubiquitous coenzymes (e.g., NADP, Coenzyme A) nor the nucleotides and their derivatives (e.g., ATP, ADP, AMP). As some of these compounds are nutrients, those are included in Supplementary Table 2.

### General Characteristics

**Figure 1:**
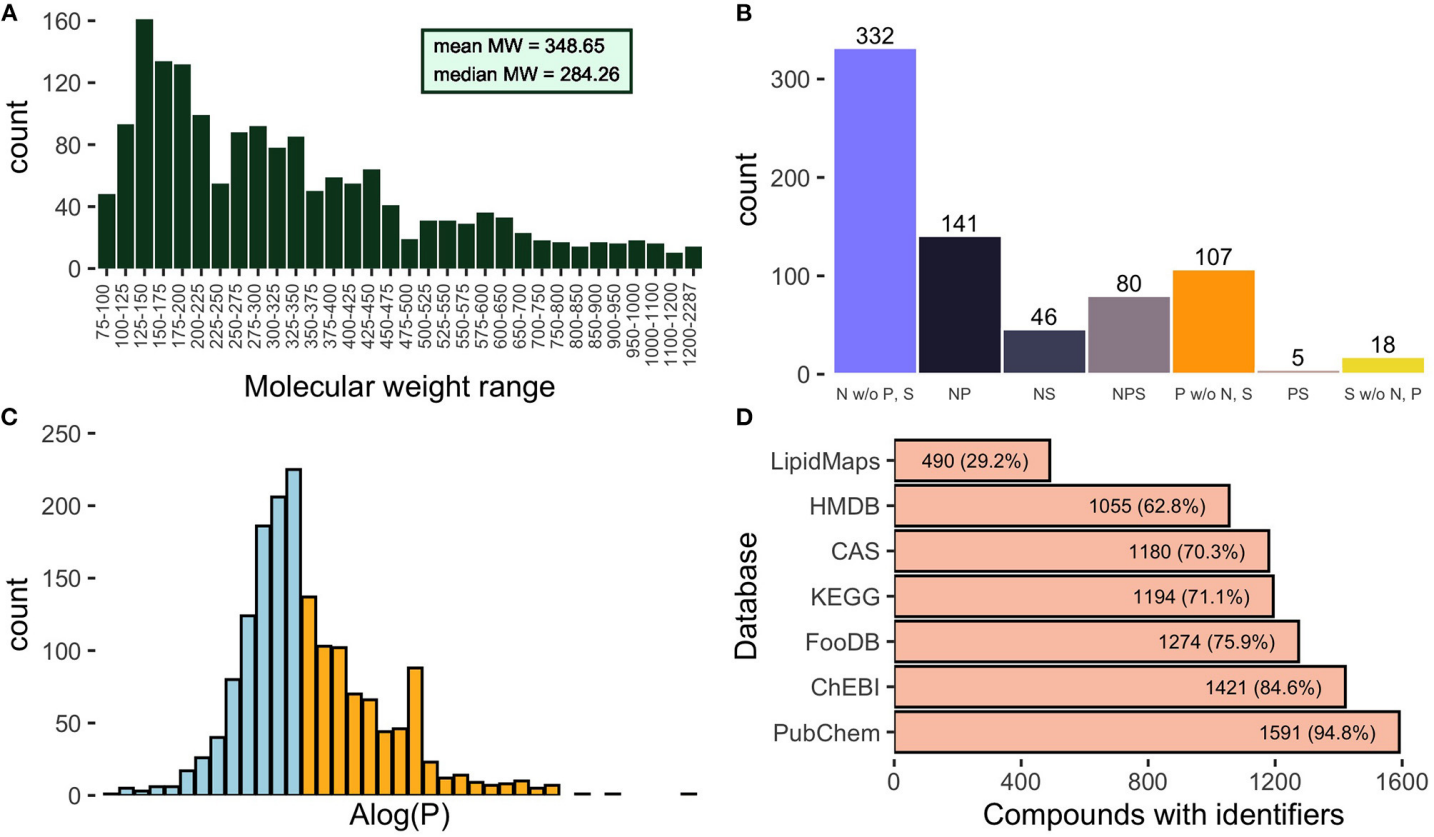
General characteristics of 1,679 naturally occurring compounds in *Citrullus lanatus*. **(A)** Distribution of molecular weights of compounds comprising the catalog. Bins from 75 to 600 are sized by 25 units, from 600 to 1,000 by 50 units, and from 1,000 to 1,200 by 100 units. **(B)** Distribution of watermelon compounds containing atoms of nitrogen (N), phosphorus (P), and/or sulfur (S). The text “w/o” indicates without, e.g., compounds without phosphorus and sulfur in the leftmost column of the plot. **(C)** Distribution of watermelon natural products at different levels of calculated hydrophilicity and lipophilicity. Molecules were counted in bins of one unit of AlogP. Those predicted to be hydrophilic are plotted in light blue with the strongest predicted hydrophilicity at the extreme left of the plot. Lipophilic compounds are in orange with the most lipophilic entities at the far right of the plot. **(D)** Compounds with identifiers in standard chemical repositories. Two hundred ninety-eight compounds have identifiers in all seven of these resources.

Molecules range in size (Figure 1A) from molecular weight 82.10 Da (dihydropyrimidine, AFC000168) to 2,286.8 Da for cold-adapted KDO2-lipid A (AFC001362). Grouping compounds into molecular weight bins of 25 units shows that molecular weight range 125–150 is the most populated with 161 compounds. The mean molecular weight in the catalog is 348.65 Da, and the median is 284.26 Da.

All molecules contain carbon except for the pyrophosphate ion (AFC000828). Oxalate (AFC00451) is the only compound that contains carbon and oxygen with no hydrogen atoms. There are 86 compounds that lack oxygen atoms, and of these, 58 are composed solely of carbon and hydrogen, ranging in molecular mass from ethenylbenzene (104.15 Da, AFC000284) to phytoene (544.94 Da, AFC000908). Additionally, 599, 333, and 149 compounds contain nitrogen, phosphorus, or sulfur atoms, respectively. Summary characteristics regarding the composition of watermelon natural products with these three atoms are presented in Figure 1B.

For each compound we determined the predicted partitioning between a hydrophobic and hydrophilic phase, using the Atomic logarithm of 1-octanol/water partition coefficient (AlogP) values (Figure 1C). This provides information on the solubility of a molecule based on its atomic constituents. A negative AlogP-value indicates a hydrophilic compound and a positive value is lipophilic. Of the natural products in this catalog, 925 (55.1%) are predicted to be hydrophilic and 754 (44.9%) lipophilic. The hydrophilicity of a compound has a direct impact on its distribution within cells and tissues and on its capacity to transit the cell membrane.

Each compound identified in watermelon is cross-referenced to the identifiers from seven different chemical compound databases (see section Materials and Methods). This information is provided to facilitate links between this resource and well-known, richly annotated databases of chemical compounds. Of the 1,679 compounds inventoried here, the range of representation spans from 1,591 (94.8%) with identifiers in PubChem (9) to 490 (29.2%) compounds found in the specialized LipidMaps (42) resource (Figure 1D). Because not all natural products cataloged here are found in the large databases and for ease of discussion, we created the AFC identifiers and assigned such to all cataloged compounds.

### Chemical Classes and Known Bioactive Compounds

**Figure 2:**
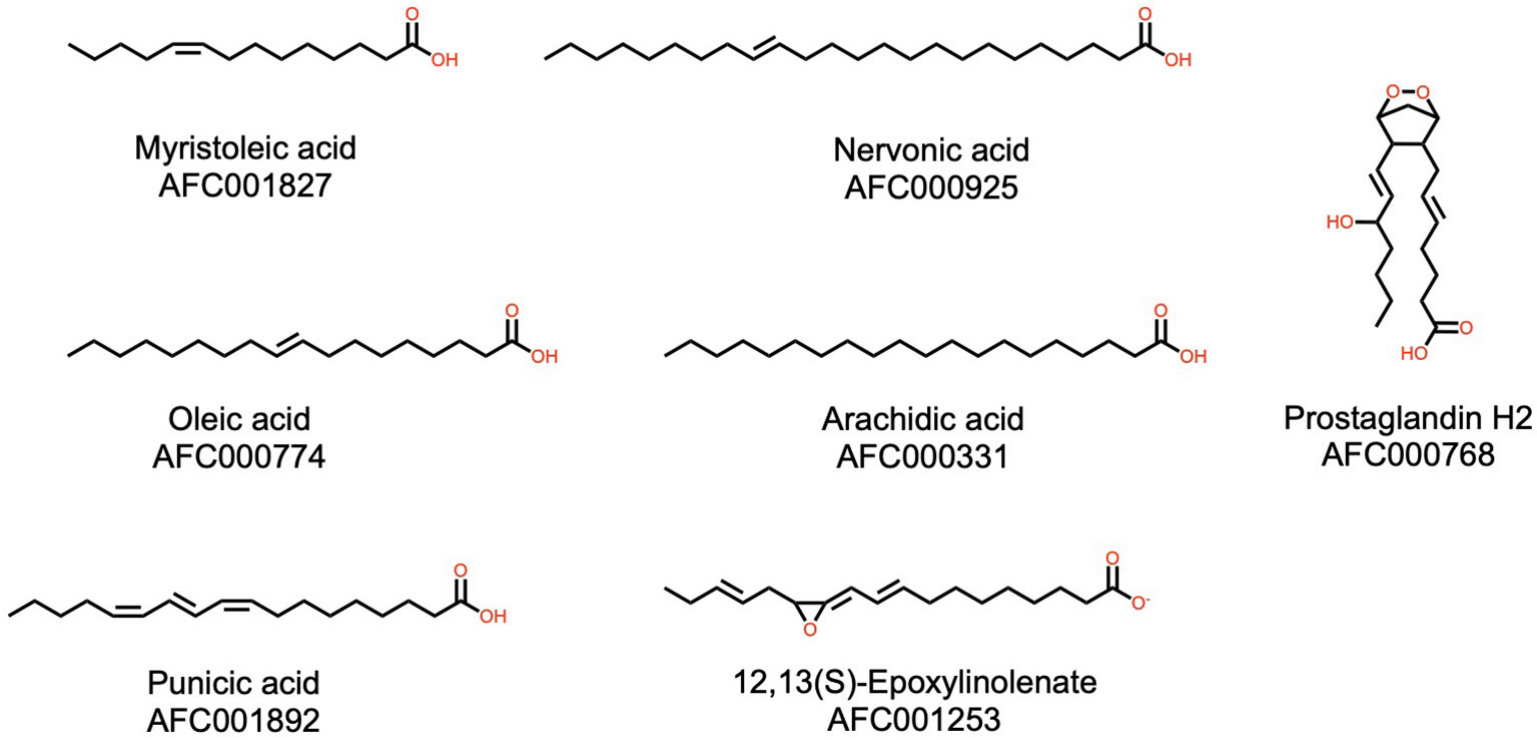
Examples of fatty acids from watermelon. Please refer to Materials and Methods (section AFC Identifiers) for the definition of the AFC identifiers.

**Figure 3:**
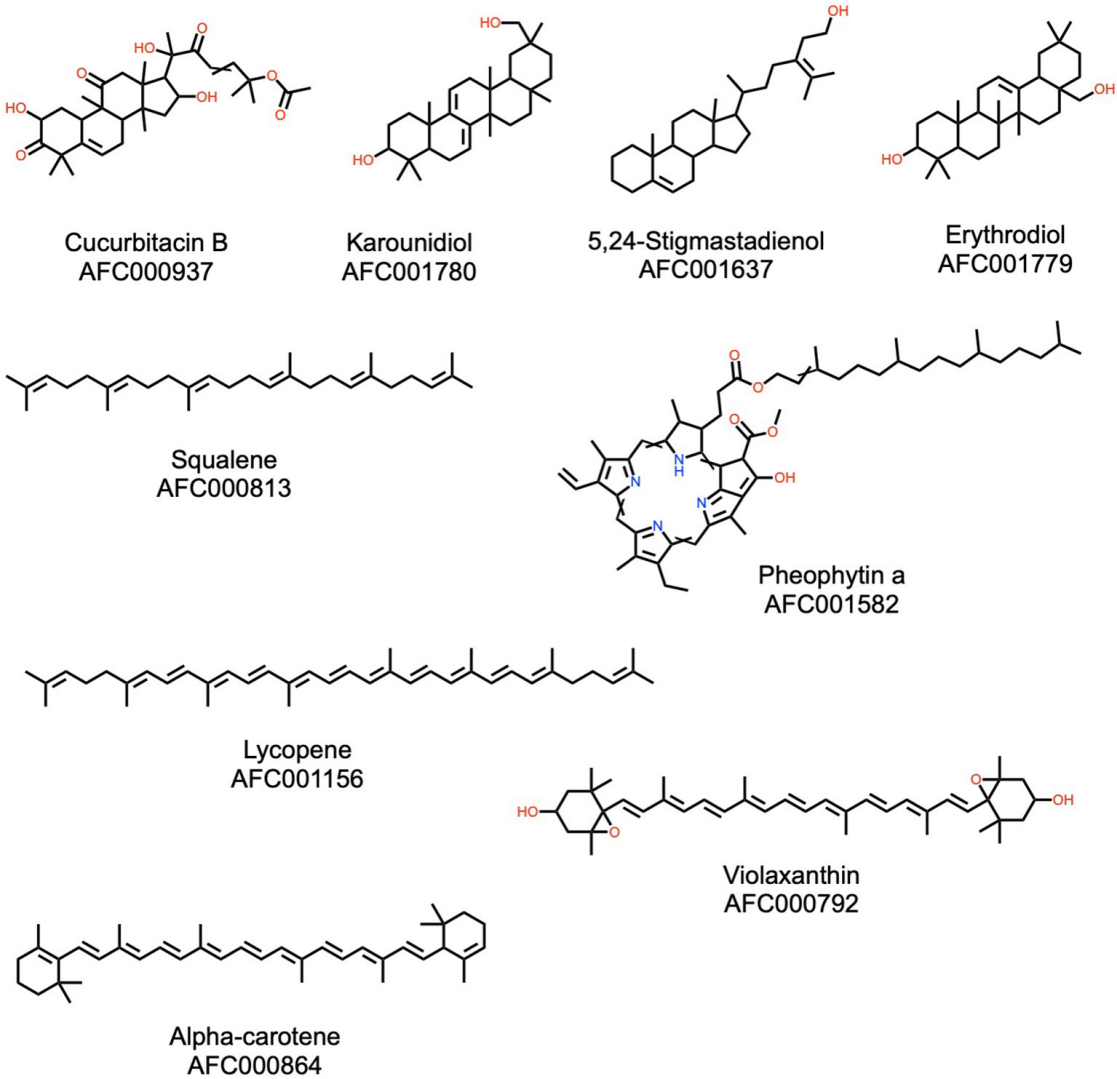
Examples of terpenoids (including triterpenoids, sterols) present in the watermelon plant. Please see Materials and Methods (section AFC Identifiers) for the definition of the AFC identifiers.

**Figure 4:**
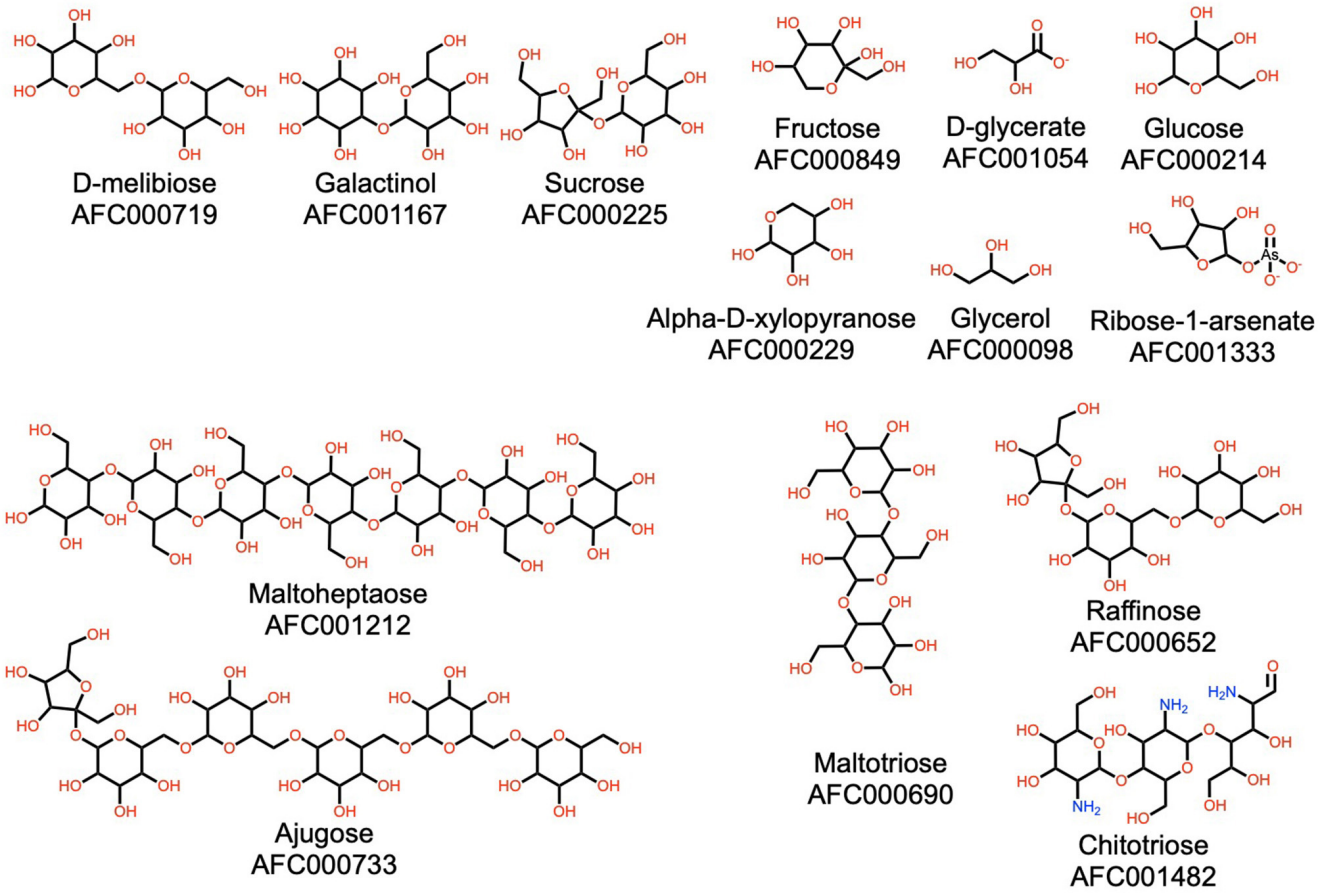
Illustration of the diversity of carbohydrates in watermelon. AFC identifiers are defined in Materials and Methods, section AFC Identifiers.

**Figure 5:**
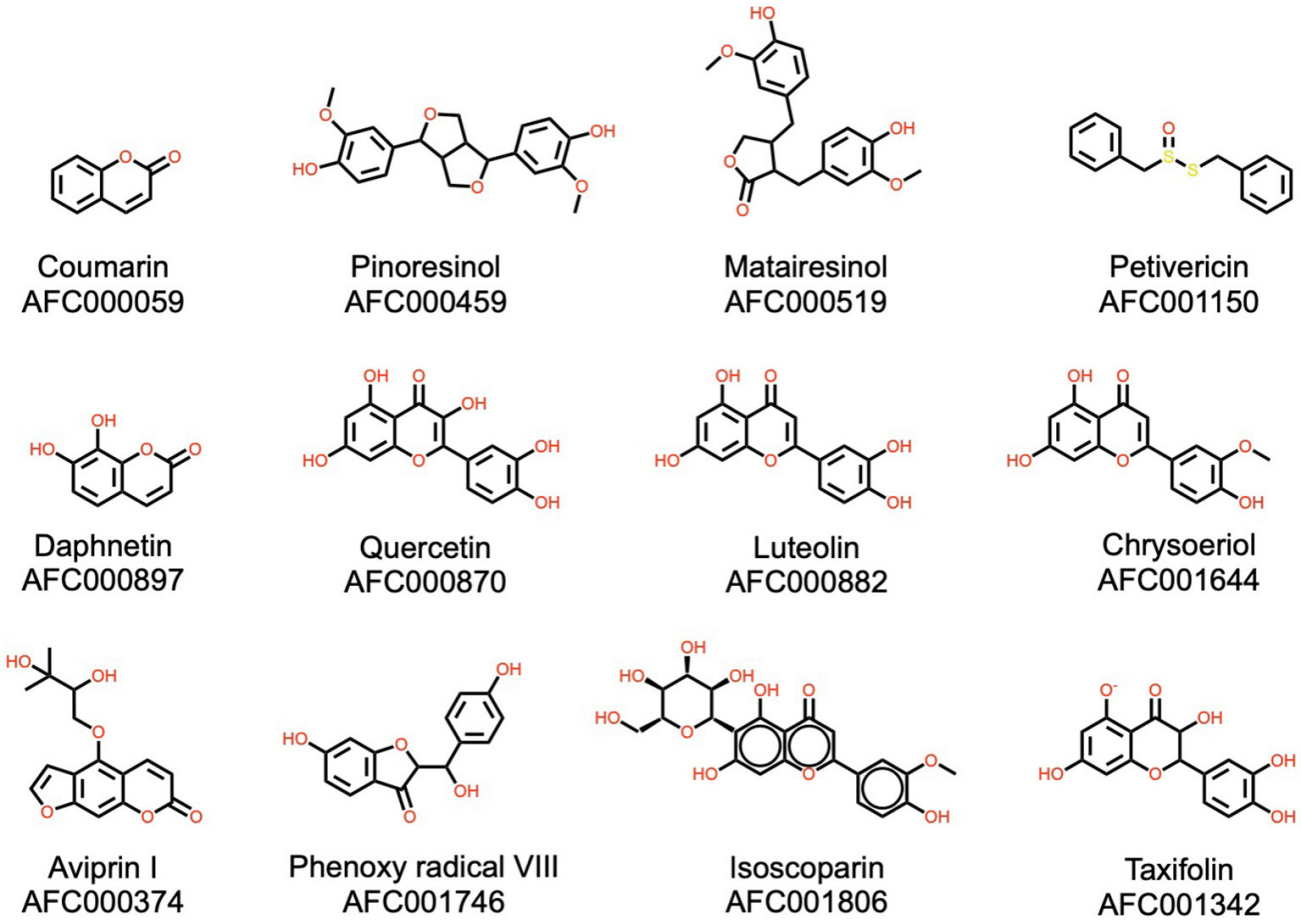
Some shikimates, phenylpropanoids, and polyphenols present in the watermelon plant, including coumarins, lignans, and flavonoids. AFC identifiers are defined in Materials and Methods, section AFC Identifiers.

**Figure 6:**
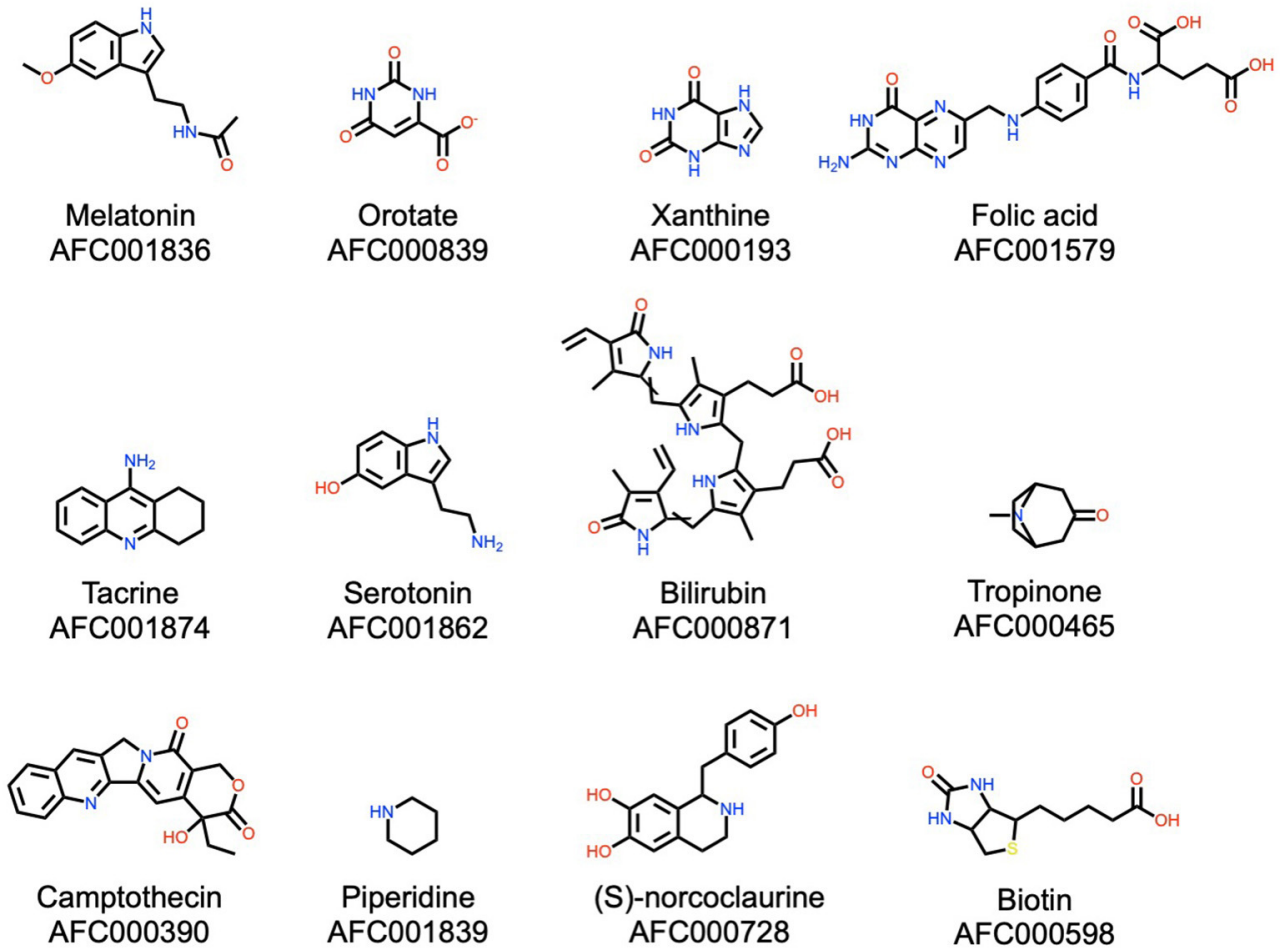
A subset of alkaloids documented in watermelon. Please see Materials and Methods (section AFC Identifiers) for the definition of the AFC identifiers.

**Figure 7:**
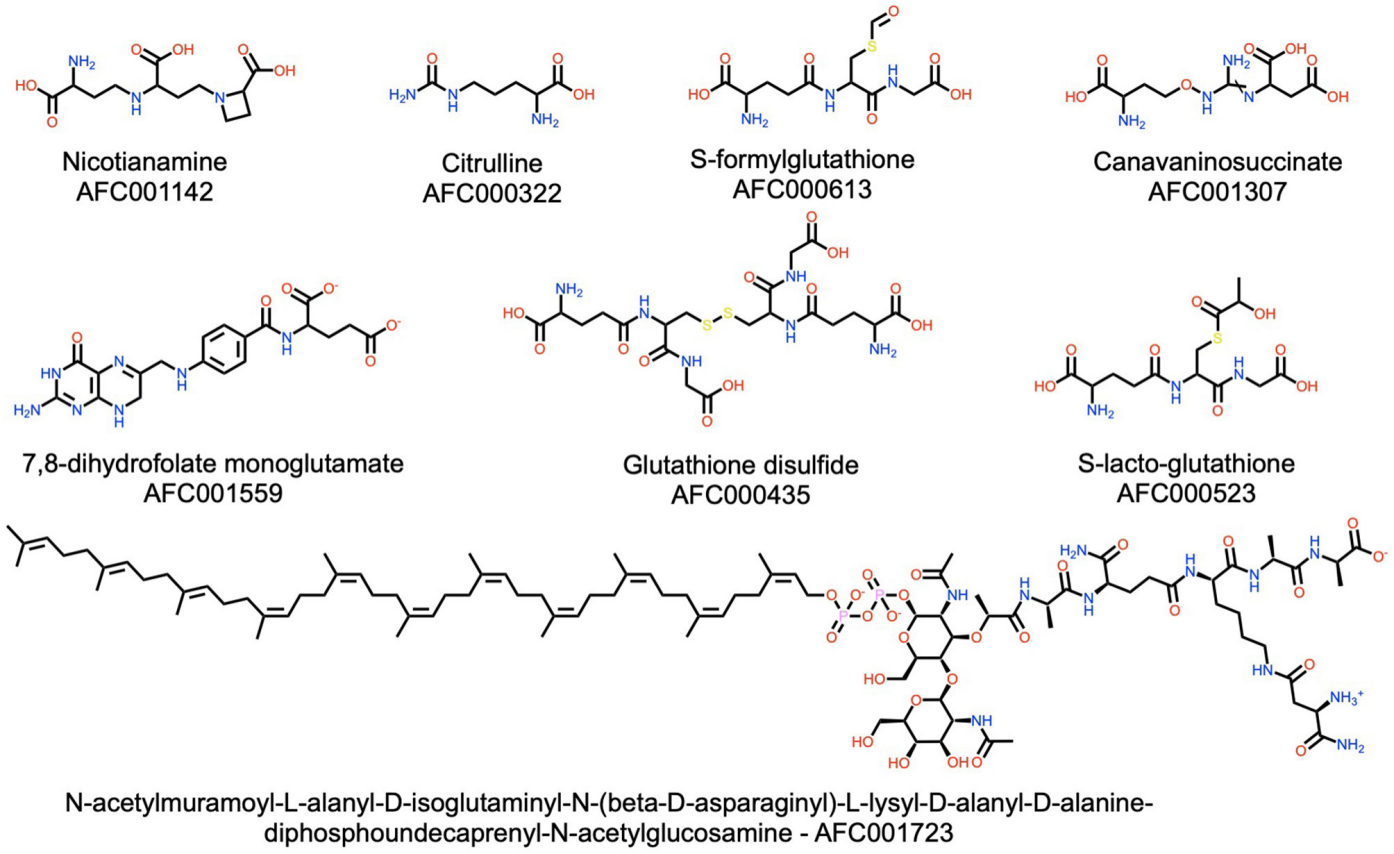
Examples of dipeptides, tripeptides, and one non-ribosomal peptide (AFC001723) present in the watermelon. Please refer to Materials and Methods (section AFC Identifiers) for the definition of the AFC identifiers.

**Figure 8:**
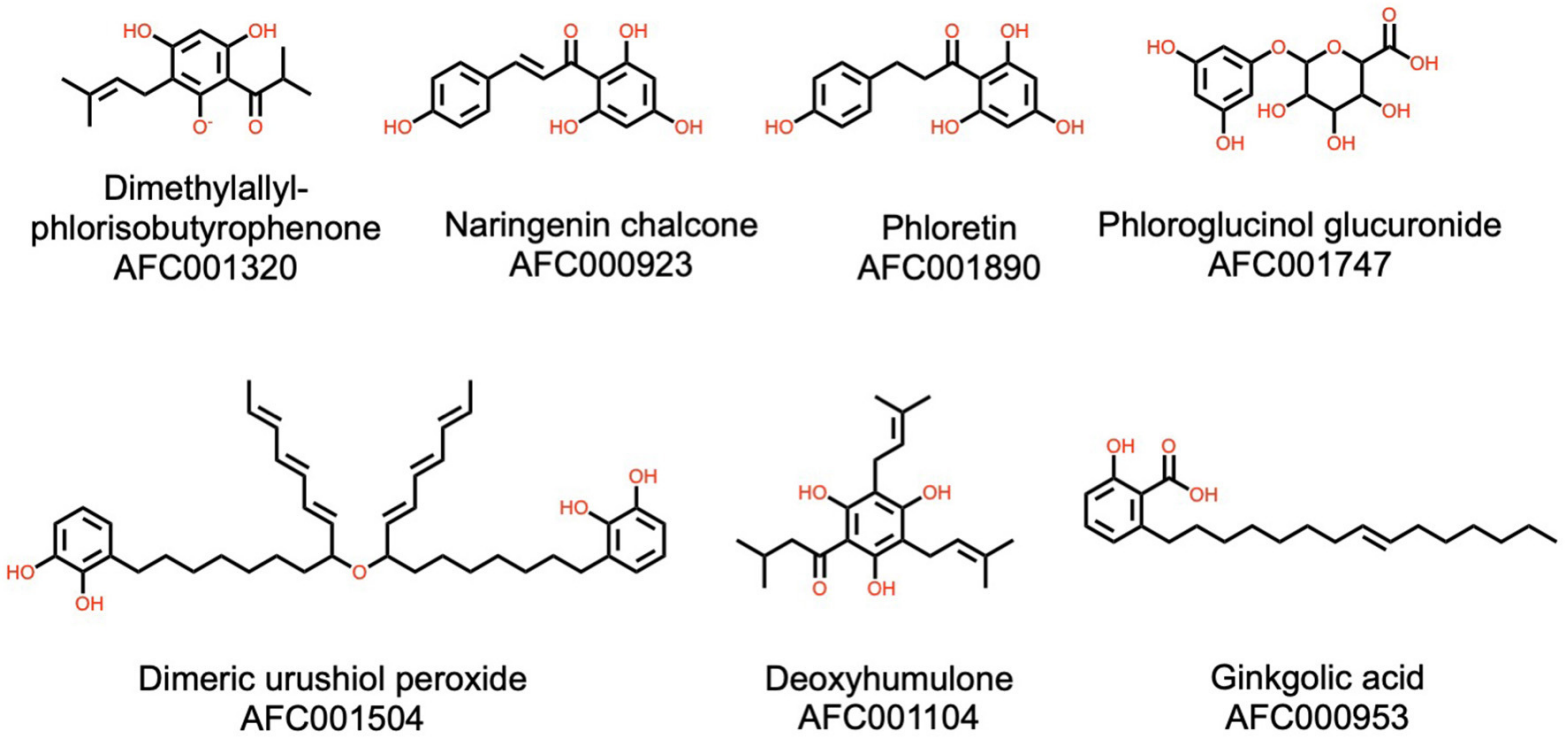
Examples of the type III polyketides, catechols, phloroglucinols, and chalcones, present in watermelon. AFC identifiers are defined in Materials and Methods, section AFC Identifiers.

The classification of watermelon compounds with NPclassifier distributed them into seven distinct chemical classes: 351 compounds were identified as fatty acids (Figure 2), 328 as terpenoids (Figure 3), 209 as carbohydrates (Figure 4), 199 as shikimates, phenylpropanoids or polyphenols (Figure 5) 142 as alkaloids (Figure 6), 101 as amino acids, peptides, and NRPs (Figure 7), and 16 as type III plant polyketides (catechols, phloroglucinols, and chalcones—Figure 8). Among the latter, 28 compounds were classified in more than one category. Lastly, 305 compounds remained unclassified because of current limitations of NPclassifier.

#### Fatty Acids

Fatty acids form a very large group of natural compounds, and are the major components of lipids. Fatty acids can be classified variously: by saturation, number of carbons, or linearity. Dietary fatty acids also are important in human health and disease prevention (50). Three hundred fifty-one fatty acids were identified in the watermelon plant (Figure 2). Of particular interest are some known functional compounds, such as nervonic acid, which is beneficial to brain function (51), oleic acid, known to be a good general anti-inflammatory (52), and punicic acid, which has a wide range of biological properties, in particular antidiabetic and anti-obesity (53). Arachidic acid was found in the seeds and is one component of nanoparticles for drug delivery (54). Interestingly, two prostaglandins are found in the watermelon plant. H2 (55) regulates dilation of blood vessels, and stimulates platelet aggregation, and E2 is involved in modulating immune responses and has anti-inflammatory activity (56).

#### Terpenoids

Terpenoids are the largest class of known natural products (57) and are characterized by their derivation from isoprene. Plant terpenoids are often used for their aromatic properties, but they also have notable pharmacological attributes. A total of 328 terpenoids have been described in watermelon, in particular cucurbitacins and carotenoids, and molecules representative of this class are shown in Figure 3. Cucurbitacins, also known as cucurbitane triterpenoids, have anti-inflammatory, antioxidant, and anticancer properties (58–60). Watermelon fruit is abundant in lycopene, which has significant antioxidant activity (61). Carotenoids as a group of phytochemicals are of intense interest for their overall benefits to human health. In particular their consumption is associated with lower risk of cardiovascular disease, cancer, and eye disease (61). Watermelon also contains squalene, a natural product with broad applications in nutrition, pharmacy, medicine, and cosmetics (62), erythrodiol, a vasorelaxant (63), and karounidiol which was observed to have anti-tumor effects (64). Distinct from terpenoids with pharmacological interest, the plant also contains pheophytin A (65), a beautiful molecule that can be used to estimate fruit ripening, and violaxanthin, which protects the plant from photooxidative damage (66).

#### Carbohydrates

The carbohydrate class of watermelon natural products is large, and despite the common association with a sweet taste, its molecules are not limited to this attribute. As expected, watermelon has a noted diversity of carbohydrates, including mono-, di-, and tri-saccharides, plus polysaccharides. Some examples of this group are illustrated in Figure 4. Only a few of these, such as glucose, fructose, and sucrose, impart a sweet taste to the fruit. The others, such as maltotriose, ajugose, and maltoheptaose, are synthesized and deposited in storage organs, such as seeds, during the maturation and ripening processes, which are then mobilized during early seed germination (67). In addition to typical carbohydrates, watermelon also contains chitotriose, an interesting carbohydrate-like molecule studied for antioxidant activity (68).

#### Shikimates, Phenylpropanoids, and Polyphenols

Shikimates, also known as shikimic acids, and the structurally similar phenylpropanoids, are a diverse family of natural products occurring in plants and synthesized from the aromatic amino acids phenylalanine and tyrosine. This family is also known for flavorful molecules, in particular flavonoids, but also coumarins and lignans. The watermelon plant contains 199 identified molecules from this chemical family (Figure 5). Among these, several have industrial or pharmacological interest, such as coumarin. Although toxic for humans in high concentrations, coumarin does add a pleasant odor in low concentrations, as is the case in watermelon. Coumarin also has a wide range of uses in industry, mainly related to its fragrance (69). Coumarin derivatives have demonstrated anti-inflammatory and antioxidant properties (70). Watermelon red flesh contains several flavonoids, polyphenols well-known for their pharmacological activities. These include luteolin, which has potential anti-cancer (71), anti-inflammatory, antioxidant, and anti-allergic activities (72), quercetin, with a vast range of activities, in particular antioxidant effects (73), and taxifolin, also recognized for its antioxidant properties (74). Watermelon also contains potent lignans, such as pinoresinol, with potential hepatoprotective effects (75) and thiosulfates like petivericin, involved in plant defense (76) with noted antibacterial and antifungal properties.

#### Alkaloids

Alkaloids are a class of natural products that contain at least one nitrogen atom and are produced by diverse organisms, with plants in particular. These molecules are known to have a wide spectrum of bioactivities, such as pharmacological applications, psychotropic, and stimulant use, and may be toxic. In general, alkaloids have a bitter taste for humans. The NPclassifier identified 142 alkaloids in the present watermelon natural products catalog, with selected examples shown in Figure 6. Among the alkaloids, particular attention is drawn to melatonin and serotonin, important for signaling and stress mitigation in plants (77), but also regulating mood, circadian cycles, and anxiety in mammals (78, 79). The watermelon fruit contains six of the eight types of water-soluble vitamin B: biotin (B7), folic acid (B9), thiamin (B1), riboflavin (B2), pantothenic acid (B5), and pyridoxine (B6). These compounds are involved in a wide range of metabolic processes in mammals and therefore are used for a broad spectrum of pharmacological applications (80). Also observed in the watermelon plant are xanthine and bilirubin, which have antioxidant effects (81, 82).

#### Amino Acids and Small Peptides

Over 100 non-proteinogenic amino acids and small peptides are reported in watermelon. The structures of selected examples are depicted in Figure 7. Among these, citrulline is most prominent, and watermelon remains its most important source known (27). Citrulline is used as a drug and in food supplements for its stimulating activity on protein synthesis in skeletal muscle (83), its cardioprotective and overall beneficial cardiovascular effects (84), and even for erectile dysfunction (85). In addition to citrulline, watermelon also contains high levels of glutathione and its derivatives (e.g., S-formylglutathione, glutathione disulfide, S-lactoglutathione), which show antioxidant activities (86). Four NRPs are reported (Figure 7) in the watermelon plant. However, as NRPs are known to be produced mainly by bacteria and fungi, caution is warranted as these NRPs might also be produced by a bacterium or fungus inside the plant or by the plant independent of bacterial or fungal infection.

#### Catechols, Phloroglucinols, and Chalcones

Sixteen natural products in the watermelon plant have been classified as catechols, phloroglucinols or chalcones, or type III polyketides produced only by plants. Representatives of this class are shown in Figure 8. Among these, two stand out for their recognized properties. Ginkgolic acid (AFC000953) is a natural product known for its anti-inflammatory (87) and neuroprotective (88) bioactivities. Phloretin (AFC001890) has various applications in medicine and cosmetics, derived from its broad and potent antioxidant activities (89).

#### Other Notable Molecules

**Figure 9:**
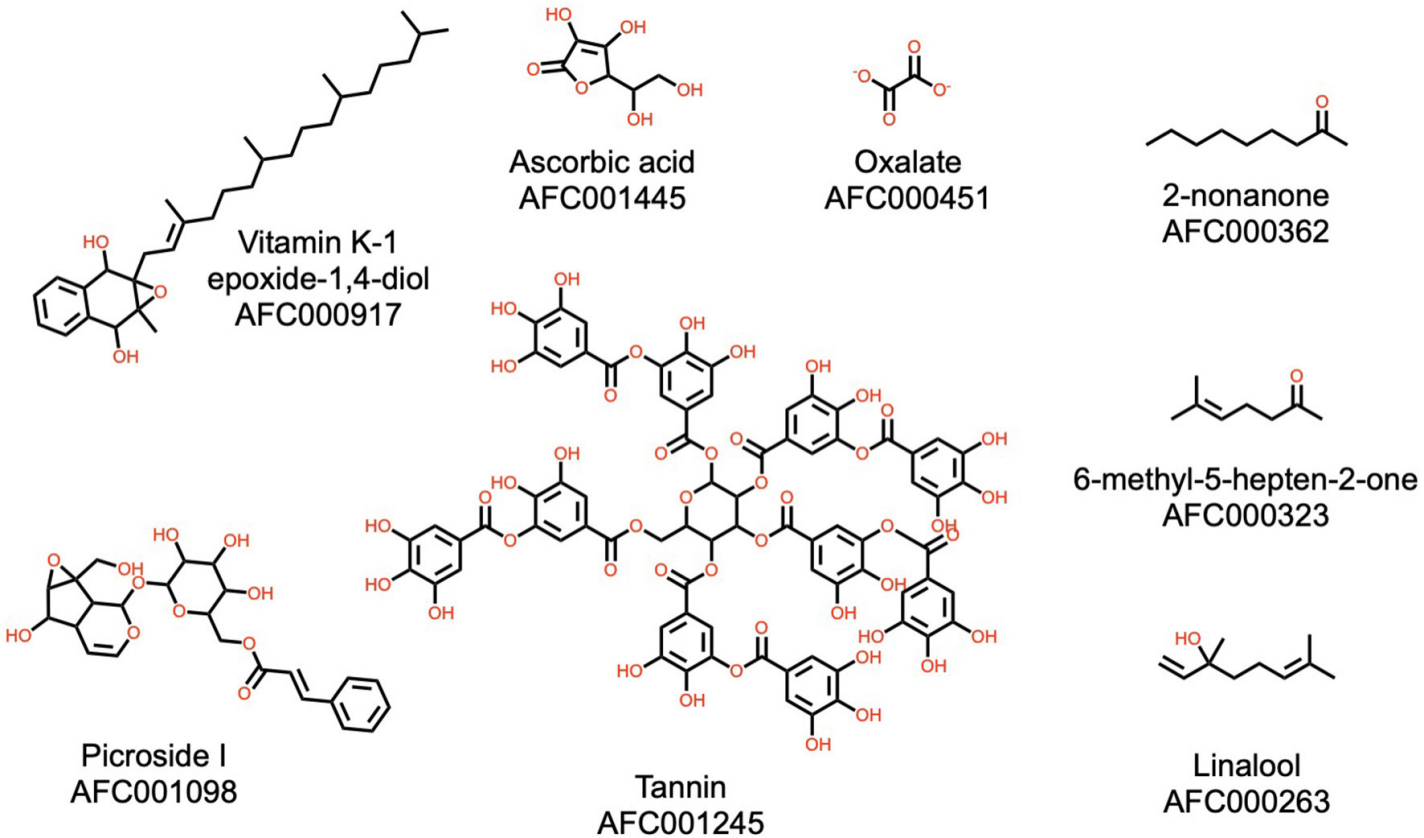
Additional noteworthy natural products present in the watermelon plant. The illustrated structures represent compounds not placed in the classes represented in Figures 2–8. These include a tannin and some volatile compounds. AFC identifiers are defined in Materials and Methods, section AFC Identifiers.

Tannins are astringent polyphenolic biomolecules widely distributed in many plant species where they are mainly involved in protection against predation (90). It therefore is not surprising to find this molecule class in the watermelon plant. Interestingly, of five compounds reported to repel the malaria mosquito *Anopheles gambiae* (91), watermelon contains three: 2-nonanone, 6-methyl-5-hepten-2-one, and linalool. Another notable compound reported in watermelon is picroside I, a potent hepatoprotective antioxidant (92, 93). Structures of these molecules are shown in Figure 9.

### Glycosylated Molecules

In addition to the aforementioned carbohydrates, watermelon contains 322 glycosylated molecules, i.e., non-carbohydrate molecules with glycosidic moieties attached. The glycosylation of a molecule positively affects its hydro-solubility and can increase or decrease its bioactivity. For example, in vitamin B6 in humans, glycosylation of the parent structure reduces its bioavailability (94).

**Figure 10:**
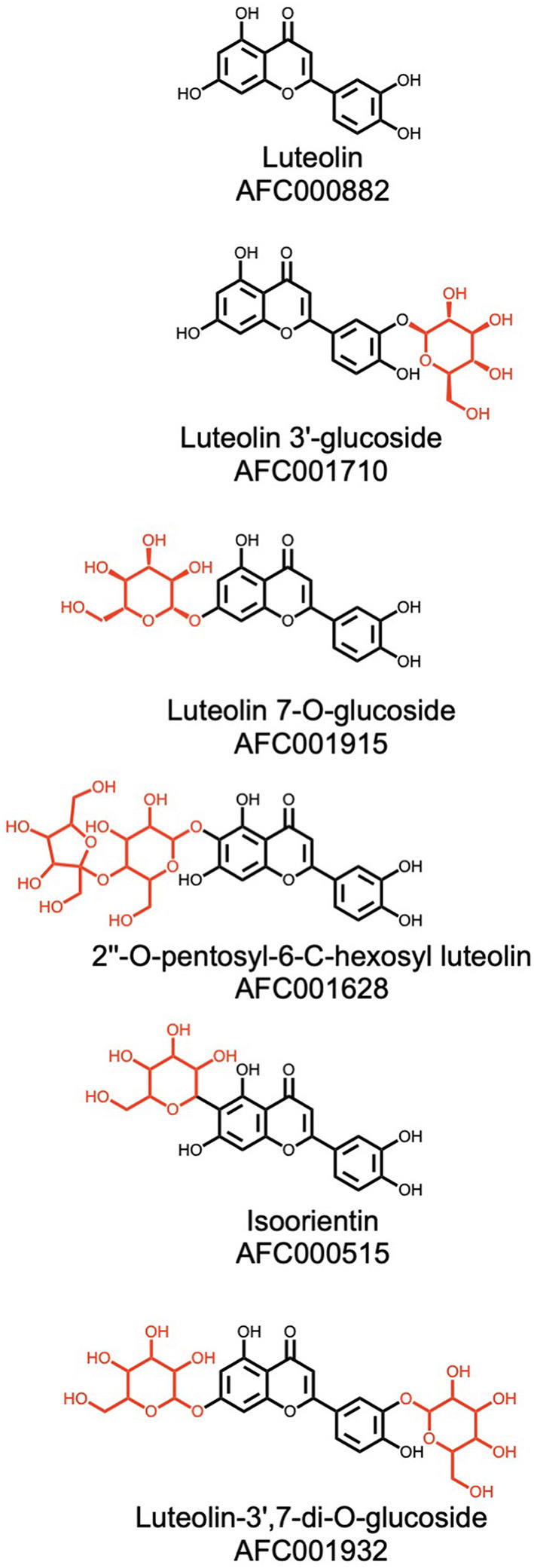
Luteolin and its glycosylated derivatives that are present in watermelon. The structure of the luteolin aglycon is depicted in black and the sugar moieties are in red. Please refer to Materials and Methods (section AFC Identifiers) for the definition of the AFC identifiers.

In watermelon, luteolin, a flavonoid with potential anti-cancer, anti-inflammatory, antioxidant, and anti-allergic activities (72, 95), has five glycosylated derivatives. In Figure 10 the sugar moieties of these derivatives are marked in red, under the luteolin aglycon. Two studies demonstrated that glycosylation of luteolin at different positions is closely linked to the intensity and modulation of its antioxidant and anti-inflammatory effects (96, 97). Such glycosylation is catalyzed *in vivo* by glycosylases, enzymes that add sugar moieties to aglycons with various selectivity. Some glycosylases add only a specific type of sugar on a specific aglycon, while others add sugars less selectively, based on aglycon substructures. Further investigation is needed to elucidate watermelon glycosylase genes and to link those enzymes to their glycosylation capabilities. Doing so will support the eventual expansion of the current catalog with other, as yet uncharacterized glycosylated natural products.

### Watermelon Chemical Space

**Figure 11:**
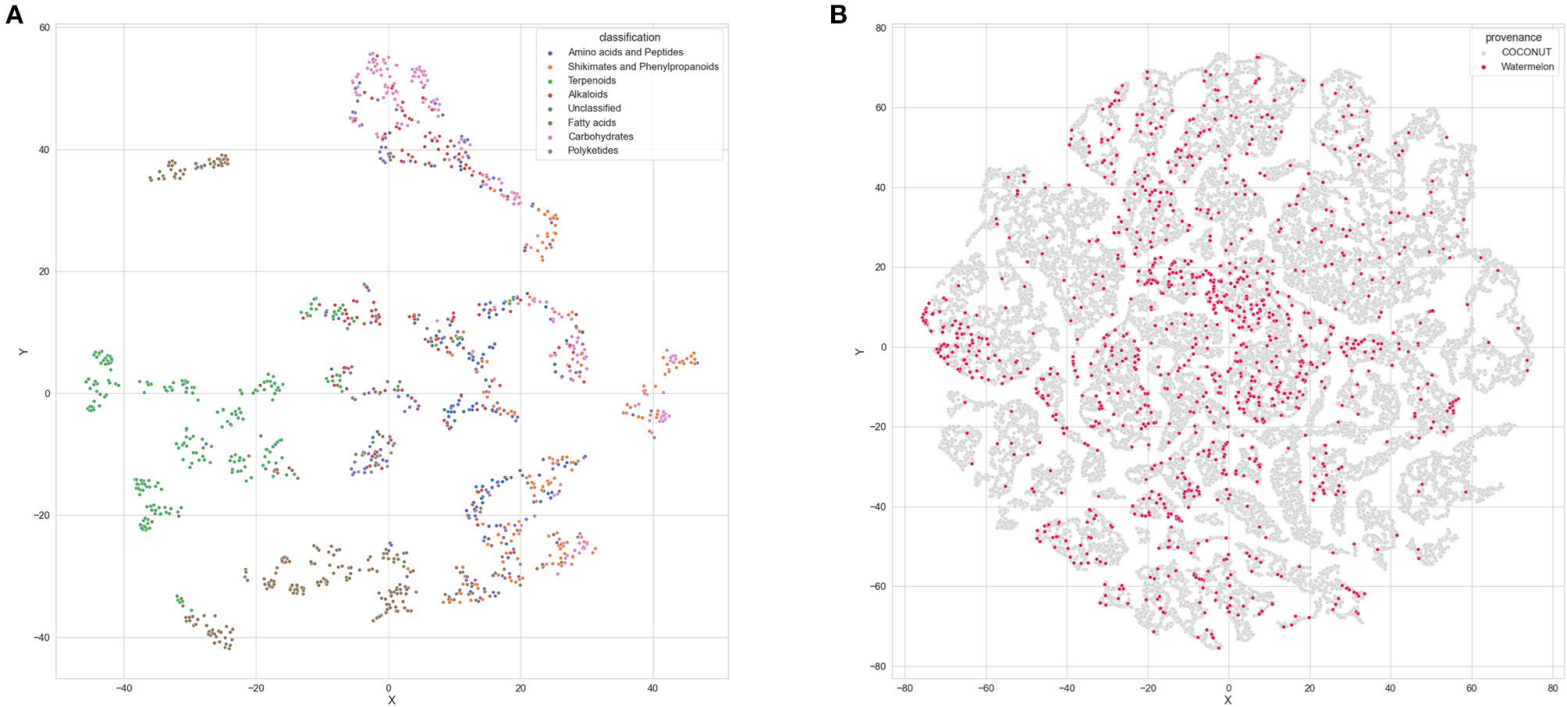
Graphical representation of the chemical space covered by watermelon natural products. **(A)** t-SNE plot of the known watermelon chemical space. Every point represents one molecule with points colored by their chemical class. **(B)** t-sne plot of the watermelon compounds (in red) within the known natural products chemical space (in gray).

t-SNE analysis, by design, converts a complex dataset of points in a high-dimensional space, such as chemical structures, and identifies an accurate representation of those data in lower-dimensional space, typically the flat 2-D version of paper or screen. Applying this tactic to the watermelon plant compound catalog shows great structural diversity (Figure 11A). The major compound classes are well-separated, as they are structurally distinct, although some map between chemical classes. The latter correspond to molecules that are hybrids, for example, glycosylated flavonoids. Compared to the chemical space occupied by all known natural products (Figure 11B, in gray), the watermelon natural products (Figure 11B, in red) cover a similar space, with only a few territories not represented. This indicates that the assembled NP catalog is relatively complete in terms of chemical diversity.

### Human Nutrition

A major impetus for assembling this list of compounds that occur naturally in watermelon was to provide information on concentration in support of research in human nutrition and plant metabolism. Moreover, in consideration of reducing food waste, different parts of the watermelon plant are considered here as these are sources of livestock feed (98, 99). Although watermelon fruit is the most popular part of the plant consumed by people, the rind and seeds are not uncommon food items. Hence, collected in Supplementary Table 2 are the levels of different compounds from different parts of the plant, as parsed from the corresponding references. Values are presented for 300 different compounds in Supplementary Table 2 and derive from various experimental conditions, different cultivars, and varieties of melon, or measurement techniques. This table also lists 1,611 other compounds that have been detected in watermelon but not quantified, including dissolved gases, nucleotides, and nucleotide derivatives, and several incompletely characterized flavone glycosides, and the like. Altogether, this table provides useful information but is intended as a guide to the source literature and nutrition databases, the latter of which may be updated in the future. We note that no data are provided for nearly 85% of the natural products tabulated.

It is well-recognized that the diet feeds the metabolism of the gut microbiota, and those metabolites generated by the microbes can affect health in humans or act as biomarkers of intake of a specific food or food group. A diverse repertoire of natural products as present in watermelon underscores its potential as a prebiotic. For example, the oligosaccharide content of watermelon, including mannitol and 1-kestose, has suggested the fruit as a source of prebiotics (100). Of different fruit peels tested, yellow watermelon showed the highest probiotic activity on *Lactobacillus rhamnosus* and *Bifidobacterium bifidum* (101). In addition, supplementing the high-fat diets of obese male mice with different watermelon products improved serum insulin and fasting blood glucose levels, as well as the hepatic metabolite profile. Furthermore, supplementation with fiber-rich extracts of rind and skin showed added improvements in glucose metabolism and energy efficiency while shifting the microbiome composition (102). Although cataloging bacterially derived metabolites is beyond the scope of this work, the catalog of natural products presented here is a necessary component that supports such efforts.

In addition to its nutrition content, watermelon is known as a folk functional food, being offered, for example, as an ethnopharmacological diuretic (103). Rat models of urolithiasis demonstrated that watermelon pulp extract reduced calcium oxalate crystal count in kidney and urine, increased urinary pH and output, elevated serum creatinine clearance, and reduced urea and creatinine levels (104). In a rat model of diuresis, watermelon pulp extract produced diuresis, reduced serum chloride levels, and elevated urinary sodium and chloride levels, in addition to inhibiting aggregation of oxalate crystals (104). Sources for these benefits include citrate, antioxidants, steroids, and alkanes. Other folk medicine uses of watermelon were for erectile dysfunction in ancient Egypt (105), as a diuretic among *Russlanddeutschen* living in Germany (103), and to quench thirst and act as a diuretic according to traditional Chinese medicine practices. In many instances, results from folk medicine, molecular nutrition, and clinical studies agree, which underscores the healthy benefits of watermelon.

### Genome Mining

The sequencing of the watermelon genome with its 11 chromosomes of different sizes is complete (21). plantiSMASH and PRISM with default parameters were used to identify eventual BGCs in each of the chromosomes (48, 49). PlantiSMASH predicted, spread across 10 of the 11 chromosomes, eight BGCs for terpene synthesis, six for saccharide synthesis, two for alkaloid synthesis, one for lignan synthesis, one for lignan-polyketide synthesis, one for saccharide-alkaloid synthesis, and three putative BGCs. Surprisingly, no NRP synthase clusters have been detected despite the documented presence of 121 NRPs in watermelon. Although the current version of PRISM is not adapted for plant genomes, it detected a total of 18 terpene BGCs across five chromosomes, overlapping significantly with plantiSMASH results for this compound category. A number of terpenes, alkaloids, NRPs, and polyketides are present in the watermelon NP catalog described here, but BGCs responsible for their synthesis were not detected by this analysis. Thus, these predictions are simply an initial glimpse of the biosynthesis capacities of watermelon. Deeper genome mining coupled with comparative genomics can lead to the discovery of other equally noteworthy natural products and the enzymes responsible for their biosynthesis.

## Summary

This catalog is a unique resource that highlights the diversity of chemical compounds in watermelon. The information presented here will be useful in crop development research integrating metabolomics, phytochemical genomics, and plant breeding to improve nutritional values of watermelon. Such a curated list of compounds associated with a single food is a necessary component in building a comprehensive catalog of natural products in all foods and can serve as a reference set for testing automated methods to capture food-compound relationships. This catalog will support detailed analyses of watermelon and can be merged with other genomics data. Such analyses can identify loci for genes whose encoded proteins facilitate synthesis, transport or storage of specific compounds, and which then can be used for crop improvement with traditional plant breeding approaches and/or biotechnology methods, constructing new links between gene, protein, and compound, and expanding existing biochemical pathways.

## Data Availability Statement

The datasets presented in this study can be found in online repositories: https://watermelon.naturalproducts.net and https://doi.org/10.15482/USDA.ADC/1522862, and in the Supplementary Material affiliated with this article.

## Author Contributions

MS, KM, ED, GM, PP-V, and LP: data extraction. MS, KM, and LP: data management and curation. MS and LP: data analysis. MS, AL, and LP: writing the manuscript. MS, KM, ED, GM, JO, PP-V, CS, AL, and LP: review and critical assessment of the manuscript. All authors contributed to the article and approved the submitted version.

## Funding

LP and JO's work was funded in part by United States Department of Agriculture project number 8050–51000-107-00D, and this entity had no part in the design of this software, collection, analysis, and interpretation of data, nor in composing the manuscript. ED and GM's work was funded by Hatch project NC02724 and USDA-NIFA-SCRI 2016-51181-25404. AL was partially supported by USDA-NIFA-SCRI, Grant Award Number: 2020-51181-32139 (CucCAP), and by the National Watermelon Promotion Board (NWPB). MS was funded by the Deutsche Forschungsgemeinschaft (DFG, German Research Foundation)–Project-ID 239748522–SFB 1127, ChemBioSys.

## Conflict of Interest

The authors declare that the research was conducted in the absence of any commercial or financial relationships that could be construed as a potential conflict of interest.

## Publisher's Note

All claims expressed in this article are solely those of the authors and do not necessarily represent those of their affiliated organizations, or those of the publisher, the editors and the reviewers. Any product that may be evaluated in this article, or claim that may be made by its manufacturer, is not guaranteed or endorsed by the publisher.
